# Scalp and skull bone metastasis in cervical carcinoma—a rare entity

**DOI:** 10.3332/ecancer.2019.969

**Published:** 2019-10-17

**Authors:** José Fernando Robles Díaz, Adela Heredia Zelaya, Alicia Milagros Avalos Rosas

**Affiliations:** National Institute of Neoplastic Diseases, Lima 34, Av. Angamos Este 2520 Surquillo, Lima, Peru

**Keywords:** Uterine cervical neoplasms, neoplasm metastasis, scalp and skull

## Abstract

Cervical cancer is the second most common cancer in women worldwide and the first in Peru; however, metastasis to the cranial scalp is extremely rare. We present the case of a 41-year-old woman diagnosed with cervical cancer IIIB, who received treatment based on concurrent pelvic radiotherapy with chemotherapy followed by brachytherapy at the primary level with complete response, developing, at 18 months, a metastatic lesion at the scalp level without evidence of recurrence in the cervix. With the rapid growth of the metastatic lesion leading to the destruction of the cranial cap, the meninges can be observed directly, without presenting to the neurological clinic.

## Introduction

Cervical cancer is the second most common malignancy in women worldwide and the most common type of cancer in women in many developing countries, mainly in Latin America [1–3]. It occurs in the reproductive period of life, with increasing incidence from 30 to 34 years of age, and a peak at 55–65 years [4].

With cervical cancer being the most common malignancy with a frequency of 24.1% among women in Peru [5], scalp metastasis is extremely rare.

The following is an unusual case of single scalp metastasis from clinical stage IIIB cervical carcinoma treated with radical external radiotherapy.

## Case report

In March 2015, a 41-year-old woman presented with gynecorrhagia associated with pelvic pain of 3 months duration. On physical examination, a 5-cm cervical tumour was found with infiltration of the upper third of the vagina, both parameters predominantly left to the pelvic bone, with clinical stage IIIB cervical cancer being diagnosed. Cervical biopsy reported moderately differentiated non-keratinising infiltrating epidermoid carcinoma. Chest X-ray imaging and multi-slice spiral pelvic abdominal tomography (MST) showed no signs of metastasis, except mild to moderate left hydronephrosis. She received external radiotherapy at a dose of 5,000 cGy in 25 sessions in pelvic fields in anteroposterior and posterior-anterior technique concurrent with carboplatin, followed by two sessions of 600 cGy at parametrial level with teletherapy. At 5 weeks, due to good parametrial response, she received two applications of high-rate brachytherapy of 800 cGy, ending in August 2015. For 18 months, she found herself free of disease.

**Figure 1.**
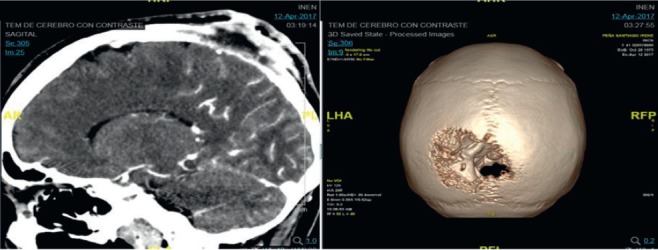
Brain MST (12.04.17): In the left parietal region, there is a solid lesion that replaces the bone marrow, measuring approximately 5.2 cm × 2 cm, infiltrating the dura mater, subcutaneous cellular tissue and adjacent skin.

**Figure 2.**
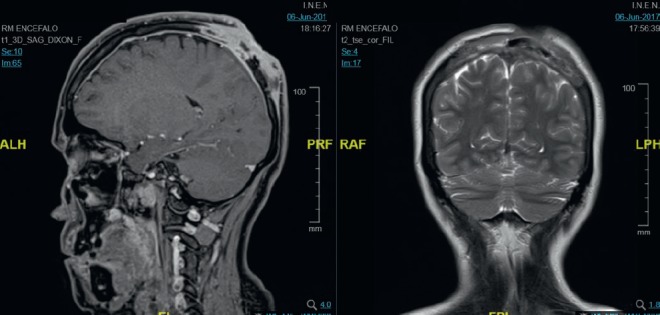
Brain MRI (06.06.17): Neoformative tissue, which infiltrates the bone marrow of the cranial calotte, in the right frontal bone, left occipital with bilateral parietal bone to left predominance, the latter infiltrates the adjacent soft parts, necrotic and ulcerated in the adjacent subcutaneous plane associated with osteolysis of the calotte, infiltrates the adjacent pachymeninges and the posterior aspect of the interhemispheric furrow, without infiltration of the parenchyma.

**Figure 3.**
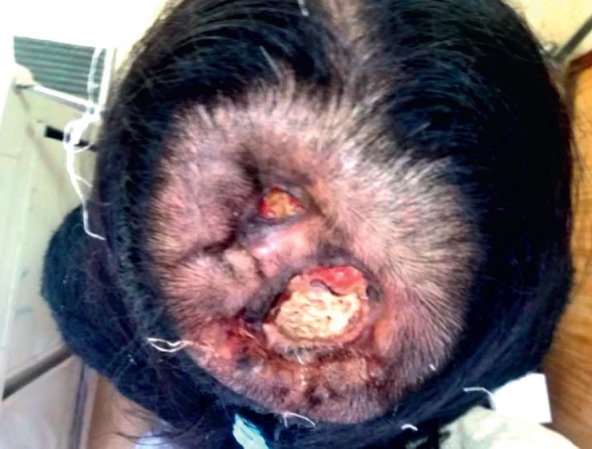
Photo of 04.07.17, with evidence of injury at the parietal level with the destruction of the cranial shell exposing the meninges.

**Figure 4.**
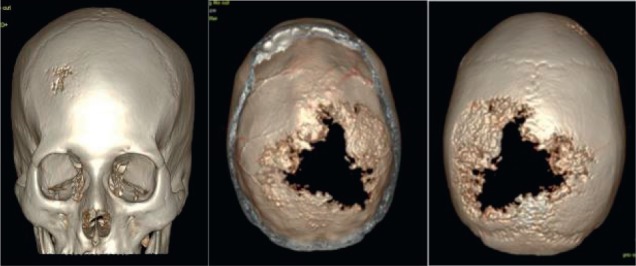
Brain MST (05.07.17): Expansive lytic lesion in the left parietal bone measuring approximately 8.37 cm × 3 cm, infiltrating the meninges. Another lesion of the same characteristics which measures 2.3 cm × 0.95 cm at the level of the right frontal bone.

In March 2017, she presented an increase in volume at the left parietal level of the scalp with an approximate size of 4 cm fixed to the bone. Brain MST was performed, indicating the presence of a solid lesion at the left parietal level, which replaced the 5 cm × 7 cm bone marrow and compromised the dura mater, subcutaneous cellular tissue and adjacent skin (Figure 1). Biopsy of the lesion reported moderately differentiated non-keratinising infiltrating epidermoid carcinoma. The patient lost her sight, and in June 2017, magnetic resonance imaging (MRI) was performed (Figure 2), showing bilateral parietal lesion with predominantly ulcerated, necrotic left that infiltrated the pachymeninges and a new lesion at the right frontal level. She was evaluated by the Department of Head and Neck Surgery, being catalogued as non-surgical. She was evaluated in the Radiotherapy department, where an ulcerated parietal level lesion with raised edges on scalp with extensive involvement was found, and with the diffuse of soft tissues with destruction of the skull of approximately 9 cm exposing the meninges at the parietal level and an elevated lesion of 3 cm at the right frontal level of hard, non-ulcerated consistency was also found (Figures 3 and 4).

**Figure 5.**
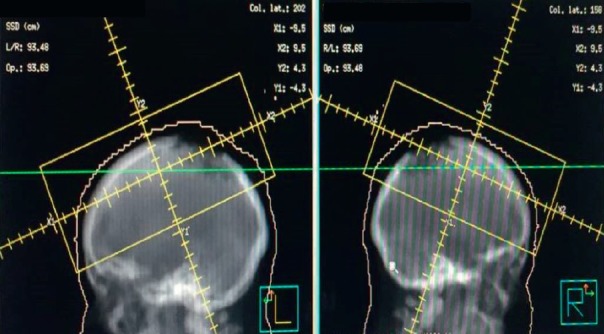
Opposite lateral treatment field covering the two lesions at 51Gy in 17 sessions with photons.

**Figure 6.**
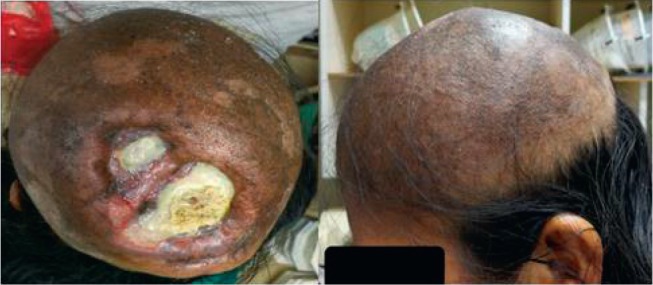
Photo of 09.08.17, 10 days after radiotherapy treatment. Borders of ulcers in reepithelialisation, no bleeding.

In a multidisciplinary meeting, it was decided to treat with radiotherapy in opposite lateral fields, covering the two lesions of the cranial cap at a dose of 5,100 cGy in 17 sessions at 300 cGy per fraction (Figure 5). Eighteen months later, she is currently under follow-up, resulting in daily healing and complete pain relief (Figure 6).

## Discussion

The spread of cervical cancer usually occurs by contiguous extension, extending from the cervix to para-cervical tissues, vaginal and parametrial tissues, to the bladder and/or rectum in advanced stages. On the other hand, it can spread through the lymphatic system spreading to the primary pelvic nodes, continuing with the common and para-aortic iliac [6]. However, the hematogenous spread is infrequent, occurring in advanced stages of the disease, especially in the lung, liver, bones and non-regional lymph nodes [1–3].

There are isolated reports of distant metastases in unusual sites such as orbit, brain, breast, heart, thyroid, kidney, spleen, intestine, muscle and scalp [7, 8], the skin of the abdomen and lower limbs are usually affected, possibly because of proximity to the pelvic region, skin involvement being considered infrequent [1, 2, 8].

Scalp metastasis is very rare, and, so far, has been previously reported in only eight studies, and suggests not being related to the initial stage of presentation as it is detected in patients treated in early stages as well as advanced (Table 1) [1, 2, 3, 7, 9–12]. The spread that is probably explained through the hematogenous pathway, as tumour plungers that reach the branches of the external carotid artery, with the consequent local implantation [2]. Of the seven cases that were managed with radiotherapy alone, Agarwal *et al* [2] reported in 2002 a case of metastatic carcinoma of cervical cancer IIIB, receiving radiotherapy of 2,000 cGy in five sessions at the scalp level with complete relief of bone pain. Also, Takagi *et al* [3] reported in 2010 a scalp metastasis with compromise of both skull tables of cervix cancer IIB, treated with radiotherapy of 4,500 cGy in 15 sessions and Vitorino *et al* [7] in 2013, handled a metastatic lesion with invasion of skull and brain by surgical resection aimed at the treatment of established intracranial hypertension and neoplastic cytoreduction, with the objective of promoting a better response to adjuvant radiotherapy.

## Conclusion

The presence of a tumour lesion at the head level in a patient with cervical cancer should be considered clinically whether it is a second primary or a metastatic tumour. Diagnostic methods should include imaging studies such as MST and MRI of the brain, which provide useful clues, making biopsy essential for this. It is important to distinguish a primary scalp tumour from a metastatic deposit, since not only does the management and prognosis of the patient change, because it is an exceptionally rare case, but the treatment is individualised, with the aim of controlling symptoms and improving the quality of life.

## Funding

This study was funded by the Radiotherapy Department of the National Institute of Neoplastic Diseases.

## Conflicts of interest

There are no potential conflicts of interest involved in this research.

## Figures and Tables

**Table 1.** Cases of scalp metastases from cervical cancer.

N	Origin	Age	EC	Histological type	GD	Primary treatment	PLE	Treatment of metastasis	Metastatic lesion control	Ref
1	Japan	59 a	IIIB	Squamous cell carcinoma	III	RT	4 m	None	Dies in 3 months	[9]
2	India	45 a	IB	Squamous cell carcinoma	I	—	—	RT	—	[10]
3	India	45 a	IIB	Squamous cell carcinoma	—	RT(50Gy/25Fx + RIC)	8 m	RT (4,500cGy/15Fx)	4 months	[1]
4	India	60 a	IIIB	Squamous cell carcinoma	II	RT(45Gy/20Fx + RIC)	2 m	RT (2,000cGy/5Fx)	—	[2]
5	Japan	45 a	IB1	Squamous cell carcinoma	III	RRHH	7 a	None	Dies in 3 months	[11]
6	India	53 a	IIA	Adenocarcinoma	II	HR + RT (28 Gy/14Fx)	4 m	RT (3,000 cGy/10Fx)	—	[12]
7	Japan	48 a	IIB	Squamous cell carcinoma small cell	—	HR + RT (45Gy/20Fx)/QT	2 m	RT (4,500 cGy/15Fx)	—	[3]
8	Brazil	55 a	IIIB	Squamous cell carcinoma	—	—	—	Resection + RT	—	[4]
9	Peru	41 a	IIIB	Squamous cell carcinoma	II	RT (50Gy/25Fx + BAT)/QT	18 m	RT (5,100 cGy/17 Fx)	18 weeks	—

Legend: EC, clinical Stage; GD, degree of differentiation; PLE, disease-free period without metastasis; Ref, literature reference; RT, radiotherapy; HR, radical hysterectomy; RIC, intracavitary radiotherapy; BAT, high-dose rate brachytherapy; QT, concurrent chemotherapy.

## References

[1] Maheshwari G, Baboo H, Ashwathkumar R (2001). Scalp metastasis from squamous cell carcinoma of the cervix. Int J Gynecol Cancer.

[2] Agarwal U, Dahiya P, Chauhan A (2002). Scalp metastasis in carcinoma of the uterine cervix – a rare entity. Gynecol Oncol.

[3] Takagi H, Miura S, Matsunami K (2010). Cervical cancer metastasis to the scalp: case report and literature review. Eur J Gynaec Oncol.

[4] Sankaranarayanan R, Ferlay J (2006). Worldwide burden of gynecological cancer: the size of the problem. Best Pract Res Clin Obstet Gynaecol.

[5] Dirección general de epidemiología. Análisis de la situación del cáncer en el Perú. http://www.dge.gob.pe/portal/.

[6] Lobo RA, Gershenson DM, Lentz GM (2017). Comprehensive Gynecology.

[7] Vitorino-Araujo JL, Veiga JC, Barboza VR (2013). Scalp, skull and brain metastasis of squamous cell carcinoma of the cervix – a rare entity. Br J Neurosurg.

[8] Bhandari V, Kausar M, Naik A (2016). Unusual metastasis from carcinoma cervix. J Obstet Gynaecol India.

[9] Shimizu I, Hayashi S, Uehara M (1983). Cutaneous metastases to the scalp from carcinoma of the uterine cervix. Arch Dermatol.

[10] Gairola M, Sharma D, Mukhopadhyay P (2000). Scalp metastasis of a uterine cervix carcinoma. Obstet Gynecol Today.

[11] Chung J, Namiki T, Johnson D (2007). Cervical cancer metastasis to the scalp presenting as alopecia neoplastica. Int J Dermatol.

[12] Abhishek A, Ouseph M, Sharma P (2008). Bulky scalp metastasis and superior sagittal sinus thrombosis from a cervical adenocarcinoma: an unusual case. J Med Imaging Radiat Oncol.

